# Multi-environment field trials for wheat yield, stability and breeding progress in Germany

**DOI:** 10.1038/s41597-024-04332-7

**Published:** 2025-01-14

**Authors:** Tien-Cheng Wang, Till Rose, Holger Zetzsche, Agim Ballvora, Wolfgang Friedt, Henning Kage, Jens Léon, Carolin Lichthardt, Frank Ordon, Rod J Snowdon, Andreas Stahl, Hartmut Stützel, Benjamin Wittkop, Tsu-Wei Chen

**Affiliations:** 1 Section of Intensive Plant Food Systems, Albrecht Daniel Thaer-Institute of Agricultural and Horticultural Sciences, Humboldt Universität zu Berlin, Berlin, Germany; 2 Department of Agronomy and Crop Science, Christian Albrechts University of Kiel, Kiel, Germany; 3 Julius Kuehn Institute (JKI), Federal Research Centre for Cultivated Plants, Institute for Resistance Research and Stress Tolerance, Quedlinburg, Germany; 4 Institute of Crop Science and Resource Conservation, Chair of Plant Breeding, University of Bonn, Bonn, Germany; 5 Department of Plant Breeding, IFZ Research Centre for Biosystems, Land Use and Nutrition, Justus Liebig University, Giessen, Germany; 6 Bundessortenamt, Hannover, Germany; 7 Institute of Horticultural Production Systems, Leibniz University Hannover, Hannover, Germany

**Keywords:** Plant breeding, Agroecology

## Abstract

Multi-environmental trials (MET) with temporal and spatial variance are crucial for understanding genotype-environment-management (GxExM) interactions in crops. Here, we present a MET dataset for winter wheat in Germany. The dataset encompasses MET spanning six years (2015–2020), six locations and nine crop management scenarios (consisting of combinations for three treatments, unbalanced in each location and year) comparing 228 cultivars released between 1963 and 2016, amounting to a total of 526,751 data points covering 24 traits. Beside grain yield, ten agronomic traits, four baking quality traits, plant height, heading date, maturity date and six fungal disease infection indices are included. Additionally, we provide management records, including fertilizer use, plant protection measures, irrigation, and weather data. We demonstrate how this dataset can address four agronomic questions related to GxExM interactions. Further potential applications of the dataset include empirical analyses, genomic and enviromic analyses for breeding targets, or development of decision-supporting models for agricultural management and policy decisions.

## Background and summary

Wheat (*Triticum aestivum* L.) is a cornerstone of the world’s food supply. Its products cover 19% of the calorie intake and 20% of the protein consumption of the world’s population. In Europe the relevance is even higher, with 25% of calorie intake and 26% of protein consumption by humans accountable to wheat (average of the years 2014-2018^1^). After a rapid and sustained global increase in wheat yields in the second half of the 20th century^2^, many countries with high wheat yields - including France, the United Kingdom and Germany - have recently experienced little to no yield progress^2–5^. Thus, the trajectory of total global food production is currently below the rate of increase needed to adequately feed the world population in 2050^6^. Consequently, significant progress in crop science and breeding is required to achieve the desired yield increases in wheat.

Improvements of genotypes and cropping systems are needed in the context of recent challenges of climate change and the parallel increase in social demand for reductions in environmental pollution, atmospheric emissions and the use of agrochemical inputs in crop production systems^7,8^. To study crop performance in terms of variation due to environmental change and genotypic improvement (breeding), multi-environmental trials (MET) are indispensable^9–13^.

**Fig. 1:**
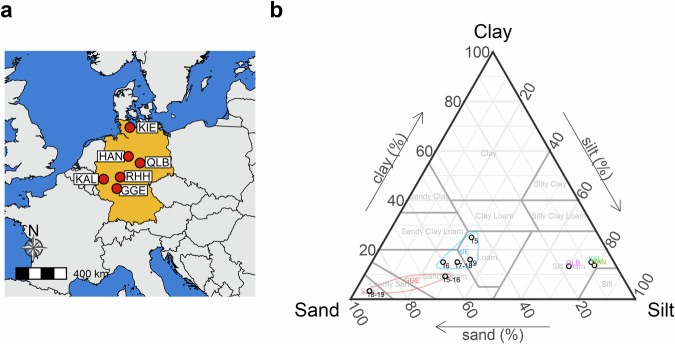
Locations and soil characteristics of six experimental fields in multi-environmental trials (MET) between 2015 and 2020. (**a**) Geographic locations. Abbreviations for six locations: Gross Gerau (GGE), Hannover (HAN), Klein Altendorf (KAL), Kiel (KIE), Quedlinburg (QLB), and Rauischholzhausen, (RHH). (**b**) Soil properties. Colours in (**b**) indicate different locations, while numbers represent experimental years. Slight variations in soil properties between years at the same location are due to field alternations within the location across experimental years.

**Fig. 2:**
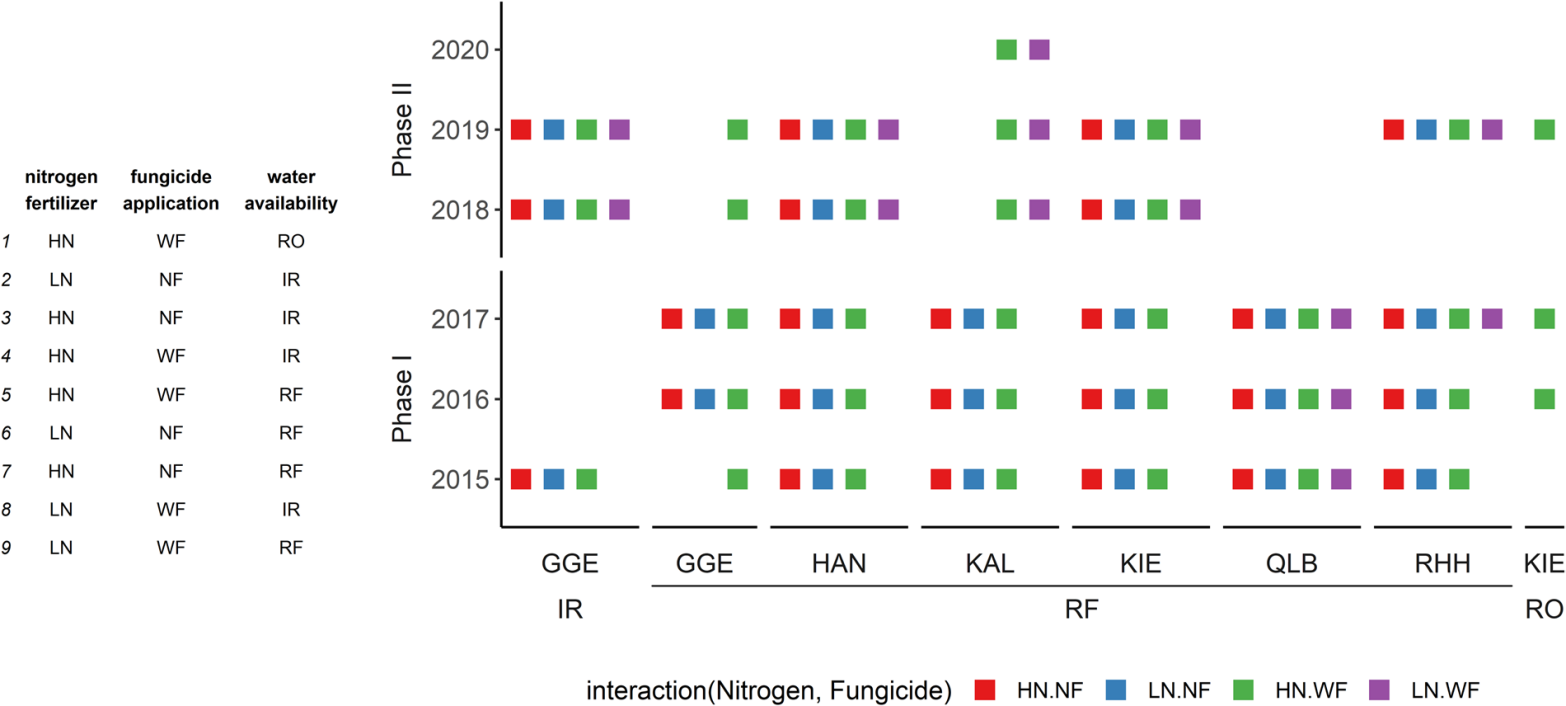
Unbalanced nine managements in multi-environmental trials (MET) dataset. Nine managements comprise of three treatments: nitrogen fertilizer, fungicide application and water availability. Nitrogen treatments has two levels: high (HN: 220 kg N ha^−1^) and low (LN: 110 kg N ha^−1^). Fungicide treatment contains two application levels: with (WF) or without (NF) fungicide application. Water availability treatment has three levels: rain-fed (RF), irrigated (IR) and rainout-shelter (RO). Abbreviations for six locations: Gross Gerau (GGE), Hannover (HAN), Klein Altendorf (KAL), Kiel (KIE), Quedlinburg (QLB), and Rauischholzhausen (RHH).

**Fig. 3:**
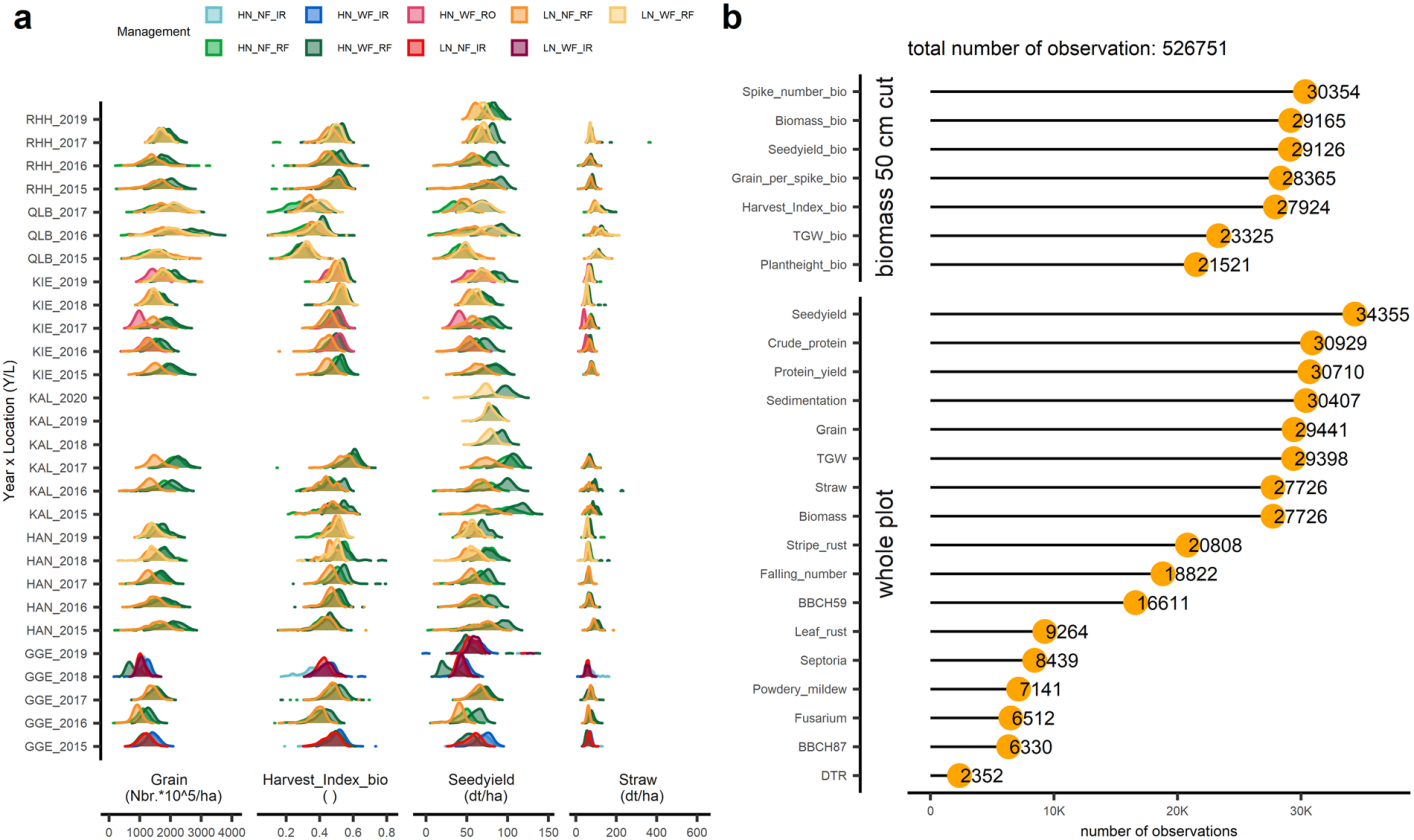
An overview of the multi-environmental trial (MET) dataset containing 24 traits of winter wheat collected across six locations in Germany (GGE, HAN, KAL, KIE, QLB, RHH) with nine managements during six years (2015–2020). (**a**) Density plot of four agronomic traits (harvest index, grain number, grain yield and straw dry mass at maturity) as examples to demonstrate the effect of managements (M) on traits distributions across 29 combinations of year by location (Y/L). (**b**) Total number of observations for 24 traits across all combinations of growing conditions (year by location by management; Y/L/M) from sampling sources collected from 50 cm cut and whole plot. Abbreviation of locations: Gross Gerau (GGE), Hannover (HAN), Klein Altendorf (KAL), Kiel (KIE), Quedlinburg (QLB), and Rauischholzhausen (RHH). Un-balanced nine managements comprise of three treatments: nitrogen fertilizer, fungicide application and water availability. Nitrogen treatments has two levels: high (HN: 220 kg N ha^−1^) and low (LN: 110 kg N ha^−1^). Fungicide treatment contains two application levels: with (WF) or without (NF) fungicide application. Water availability treatment has three levels: rain-fed (RF), irrigated (IR) and covered with rainout-shelter (RO).

The unique MET dataset presented here combines 29 environments (unbalanced combinations among six years and six locations), 9 agricultural management scenarios (unbalanced combinations among three treatments, depending on the combination of year and location), and 228 genotypes (released cultivars) with detailed field phenotyping of 24 labour-intensive traits (e.g. total final biomass) of winter wheat (*Triticum aestivum* L.). The MET were conducted for six years (2015–2020) in Germany at six locations (Fig. 1): Gross Gerau, Hannover, Klein Altendorf, Kiel, Quedlinburg, and Rauischholzhausen. The management scenarios comprised three treatments (Fig. 2): nitrogen treatment with two total fertilizer levels (HN and LN, with 220 and 110 kg N ha^−1^, respectively), fungicide treatment with (WF) or without fungicides (NF) and a water availability treatment with three levels: irrigated (IR), rain-fed (RF) and rain-out shelter (RO). All MET were conducted with a panel of 228 cultivars released between 1963 and 2016. In total, there are 526,751 data points for 24 traits, including grain and biomass yield, agronomic traits, grain quality traits and fungal disease infection scores (Table 1 and Fig. 3).

**Table 1:** Names, sampling source, column name range and unit of 24 traits.

trait full name	trait source	trait name in dataset	trait range	unit
above-ground dry mass at maturity	50 cm cut	Biomass_bio	0~3495	g/m2
harvest index	50 cm cut	Harvest_Index_bio	0.1~0.79	
grains per spike	50 cm cut	Grain_per_spike_bio	3.6~144.2	number
plant height	50 cm cut	Plantheight_bio	40~145	cm
grain yield	50 cm cut	Seedyield_bio	28.3~1815	g/m2
spike number	50 cm cut	Spike_number_bio	48~1390	number /m2
thousand grain weight	50 cm cut	TGW_bio	4.7~77.8	g
day when 75% of the ears are visible	whole plot	BBCH59	123~181	days of year
day when 75% hard dough	whole plot	BBCH87	175~213	days of year
above-ground dry mass at maturity	whole plot	Biomass	14.2~732.8	dt/ha
crude protein percentage per grain dry mass	whole plot	Crude_protein	6.2~21.3	%
leaf tan spot caused by *Drechslera tritici-repentis*	whole plot	DTR	0~100	% leaf area
falling number	whole plot	Falling_number	60~700	s
fusarium head blight	whole plot	Fusarium	0~27	% spike
number of grains per unit area	whole plot	Grain	143.7~3915.5	number x 10^5^/ha
leaf rust caused by *Puccinia triticina*	whole plot	Leaf_rust	0~90	% leaf area
powdery mildew caused by *Blumeria graminis* f. sp. *tritici*	whole plot	Powdery_mildew	0~100	% leaf area
grain protein yield	whole plot	Protein_yield	0~22.2	dt/ha
sedimentation	whole plot	Sedimentation	2.1~83.3	ml
grain yield	whole plot	Seedyield	0~141.6	dt/ha
leaf spot caused by *Septoria tritici*	whole plot	Septoria	0~80	% leaf area
above ground biomass subtracted by grain yield	whole plot	Straw	8.9~625.4	dt/ha
stripe rust caused by *Puccinia striiformis*	whole plot	Stripe_rust	0~100	% leaf area
thousand grain weight	whole plot	TGW	11.9~67.4	g

Parts of this MET dataset have been used to demonstrate that: (1) long-term breeding has improved grain yield in European winter wheat independently of input intensity among management scenarios^10^; (2) traits for sink and source capacity are co-selected throughout breeding history^14^; (3) stimuli affect the formation of yield components in a cultivar- and stage-specific manner^11^; (4) the correlation of determination between traits and year of release^13^; and (5) breeding progress in fungal disease resistance has contributed to breeding progress in yield^12^. Here we present the complete MET dataset, including previously unpublished results, and further showcase the value of this dataset for studying GxExM interactions by answering four research questions: (1) How consistent are agronomic traits between years (Y), locations (L) and managements (M) and how does the combination of Y, L and M affect trait consistency? (2) Can a well-calibrated crop model that considers GxExM interactions properly represent the trait-trait correlations observed in the fields? (3) To which extent has breeding progress in agronomic traits contributed to breeding progress for grain yield? (4) How do individual agronomic traits contribute to yield stability?

## Methods

### Overview of MET dataset: 228 genotypes and 100 growing conditions (Y/L/M)

The MET dataset was collected with the support of the project Breeding Innovations in Wheat for Efficient Cropping Systems (BRIWECS). Experiments were conducted in Germany from 2015 to 2020 in six locations (Fig. 1 and Fig. 2), including Gross Gerau (GGE), Hannover (HAN), Klein Altendorf (KAL), Kiel (KIE), Quedlinburg (QLB) and Rauischholzhausen (RHH).

Management scenarios comprised three treatments with different levels of nitrogen fertilizer, fungicide application and water availability (Fig. 2). Nitrogen fertilizer treatment includes two application levels: high (HN: 220 kg N ha^−1^) and low (LN: 110 kg N ha^−1^), both include soil mineral nitrogen (0–90 cm) measured in early spring. Fungicide treatment contains two application levels: with (WF) or without (NF) fungicide application. Water availability treatment has three levels: rain-fed (RF), irrigated (IR) and rainout-shelter treated (RO). Most of the managements were grown under the rain-fed. Only Gross Gerau and Kiel were additionally tested with irrigated and rainout-shelter treatments, respectively. The aim of the water availability treatments in these two locations was to compare them with the main on-farm practice (HN_WF_RF). In Gross Gerau from a subset of seasons (2015, 2018, and 2019), all managements were irrigated, together with only one rain-fed treatment for high nitrogen with fungicide application. In Kiel, all managements were grown under rain-fed, along with a management HN_WF treated with rainout-shelter in a subset of seasons (2016, 2017, and 2019).

The MET dataset contains a panel of 228 winter wheat cultivars, with year of release ranging from 1963 to 2016. The BRIWECS project was conducted in two phases. In Phase I^10–14^, experiments followed a randomized block design (2015–2017) with 220 cultivars (except for Rauischholzhausen in 2017). In Phase II^10^, experiments followed a full treatment-factorial design (2018-2019) with 52 selected cultivars that were a subset of the 220 cultivars from Phase I. Note that each cultivar has two replicates for each treatment, except for rain-fed treatments (2015–2019) and rainout shelter treatment (2019) in Kiel, which has three replicates.

### Dimensions and phenotypic space of the MET dataset: 24 agronomic and physiological traits

The MET dataset contains in total 526,751 observations after removing outliers (Fig. 3). There are two sample sources in the dataset: a 50 cm cut and whole plot (Table 1). A 50 cm cut sampling was collected from a non-border row of 50 cm from the whole plot at the time of grain maturity (BBCH87) to determine biomass, grain yield, thousand grain weight (TGW), spike number, plant height, grain per spike, and harvest index. Whole plot was evaluated non-destructively during the growing periods and destructively maturity. Non-destructive measurements include: heading date (BBCH59), grain maturity date (BBCH87) and six fungal diseases infection area throughout the cropping cycle (leaf tan spot, Fusarium head blight, leaf rust, powdery mildew, Septoria, stripe rust). Destructive measurements include dry mass of shoot, dry mass of straw, harvest index, grain yield, TGW, grain protein, grain falling number, grain sedimentation. Harvest index was calculated as the grain yield divided by the above ground dry mass at maturity. For details of trait collections see material and methods from^10^.

Fungal disease infection (% area) was recorded in the field for each plot with visual infection score ranging from 0-100^12^. Disease scores were collected from natural infections in the field, except for the management scenarios HN_NF_RF and LN_NF_RF in Quedlinburg, where manual inoculation was applied with pathogens of stripe rust, leaf rust and Fusarium head blight (for details see^12^). Total fungal infection area (TFI) is defined as the sum of infected area from all six fungal diseases, assuming that infection scores are additive:

1 $${\rm{TFI}}={\rm{stripe\; rust}}+{\rm{Septoria}}+{\rm{powdery\; mildew}}+{\rm{leafrust}}+{\rm{leaftan\; spot}}+{\rm{Fusarium\; head\; blight}}$$

### Outlier elimination

After quality control of the raw data, negative values or values with unrealistic ranges (e.g., grain yield > 3000 dt/ ha; TGW > 80 g/1000 grain) were re-called as “not available” (NA). For each growing condition, a cultivar with a trait value (HI and spike number) beyond the range of mean plus and minus four times standard deviation was considered an outlier and excluded from further calculations. After pre-processing, a total of 526,751 data points were available (Fig. 3).

### Calculation of the best linear unbiased estimates (BLUEs) of cultivar means

To provide an unbiased estimation of trait performance of each genotype under each combination of year, location and management (referred to as growing condition), BLUEs values were used for the further validation (technical validation I, III and IV, see the next sections). The MET dataset contains two experimental phases: randomized block design for phase I (2015–2017; except for Quedlinburg and Rauischholzhausen in 2017) and full treatment-factorial design for phase II (2018–2020; except for Gross Gerau, Klein Altendorf and Rauischholzhausen in 2018). For each combination of year, location and management, we included random effect from both row and column to consider the potential uneven gradient of soil fertility in the field. The calculation of BLUEs was based on the following model:

2 $${y}_{{irc}}=\mu +{g}_{i}+{R}_{r}+{C}_{c}$$

where *y*_irc_ is the performance of the *i*^th^ cultivar of the *r*^th^ row and the *c*^th^ column, *μ* is the general mean, *g*_i_ is the fixed effect of the *i*^th^ cultivar, *R*_*r*_ is the random effect of the *r*^th^ row and *C*_*c*_ is the random effect of the *c*^th^ column. Fixed effects are denoted by lowercase letters, while random effects are denoted by uppercase letters.

## Data Records

### Data storage

The data set^15^ is deposited on Figshare (10.6084/m9.figshare.27910269). There are six folders in the main directory: data, docs, figure, metadata, output and scripts. The folder data contains the raw data with three subfolders: locations, management and weather. The folder metadata contains the cultivar information on the cultivars investigated (BRIWECS_BRISONr_information.csv) and units used to describe all traits (Unit.xlsx). Folder scripts contains four files as follows: File data_cleaning.R combines files in folder *data* and remove outliers and store output (BRIWECS_data_publication.csv) in folder *output*. File extract_management.R combine files in sub-folder management in folder *data* and store four combined managements files-disease_record.xlsx, fertilizer.xlsx, plant_protection.xlsx and soil.xlsx in folder *output*. File data_overview.qmd generates visualizations showing the distributions and correlations of trait performance among different growing conditions (Y/L/M), sowing dates, precipitation and global radiation levels in each growing condition, with all relevant files stored in folder *docs*. Parts of the MET dataset have been published in previous studies (Table S1), including SNP data^10,14^, climatic data^11,16^ and adjusted means for pathogen infections^12,17^.

## Technical Validation

### Subset for technical validation I-IV

For further validation, a subset from the MET dataset (Table 2) was utilised. The subset included 15 traits in 220 genotypes growing in 45 growing conditions (year by location by management; Y/L/M), comprising three years from phase I (Y: 2015–2017), five locations (L: GGE, HAN, KAL, KIE, QLB), and managements from three rain-fed conditions (M: HN_WF_RF, HN_NF_RF, LN_NF_RF) for the technical validation I-IV. This subset is more balanced in number of genotypes and Y/L/M combinations ensuring comparability.

**Table 2:** Trait names and abbreviations for examples analyses I-IV.

full name	abbreviation	Fig. 4	Fig. 5	Fig. 6	Fig. 7 Fig. 8	Fig. 9
		R^2^_sma_	R^2^_sma_	trait-trait correlation	BP	SI
aboveground dry mass at maturity	SDM	v				
straw dry mass at maturity	Straw	v		v	v	v
flowering time	FT			v		
grain number	GN	v		v		
grain protein concentration	GP	v		v		v
grain per spike	GpS	v			v	v
grain yield	GY	v	v	v	v	v
harvest index	HI	v		v	v	v
total fungal disease infection area	TFI					v
light extinction coefficient	k			v		
leaf area index	LAI			v		
maturity	MT			v		
radiation use efficiency	rue			v		
spike number	SN	v			v	v
thousand grain weight	TGW			v	v	v

### GxExM was most prominent in straw dry mass and spike number

First validation shows the consistency of trait performance (i.e., BLUEs) across growing conditions (Y/L/M). Here, we define trait consistency (R^2^_sma_) as R^2^ derived from standardized major axis (SMA)^18,19^ regression of BLUEs of a population (220 genotypes) between two growing conditions. SMA regression assumes the source of error coming from both dependent and independent variables, therefore suitable for non-causal relationships^20^. In this validation, we demonstrate R^2^_sma_ of nine agronomic traits (Table 2), including grain yield (GY), harvest index (HI), straw dry mass (Straw), above ground dry mass (SDM), grain number (GN), grain protein concentration (GP), grain per spike (GpS), spike number (SN) and thousand grain weight (TGW).

To further validate the effect of year, location or management on R^2^_sma_, trait consistency was analysed by single or double grouping of growing conditions. Single grouping considers only year, location and management individually. For instance, when grouping by management, we calculate R^2^_sma_ between every pairs of Y/L under the same management level (e.g., HN_WF_RF). We showcased the results of two double groupings: management-location (to examine inter-years’ similarity) and management-year (to examine inter-locations similarity). One-way analysis of variance (ANOVA) was performed to examine the mean difference of R^2^_sma_ between levels within each group. Fisher’s least significant difference test was used to differentiate the mean of levels within each group once significance of ANOVA (*p*-value < 0.05) was detected for the group.

**Fig. 4:**
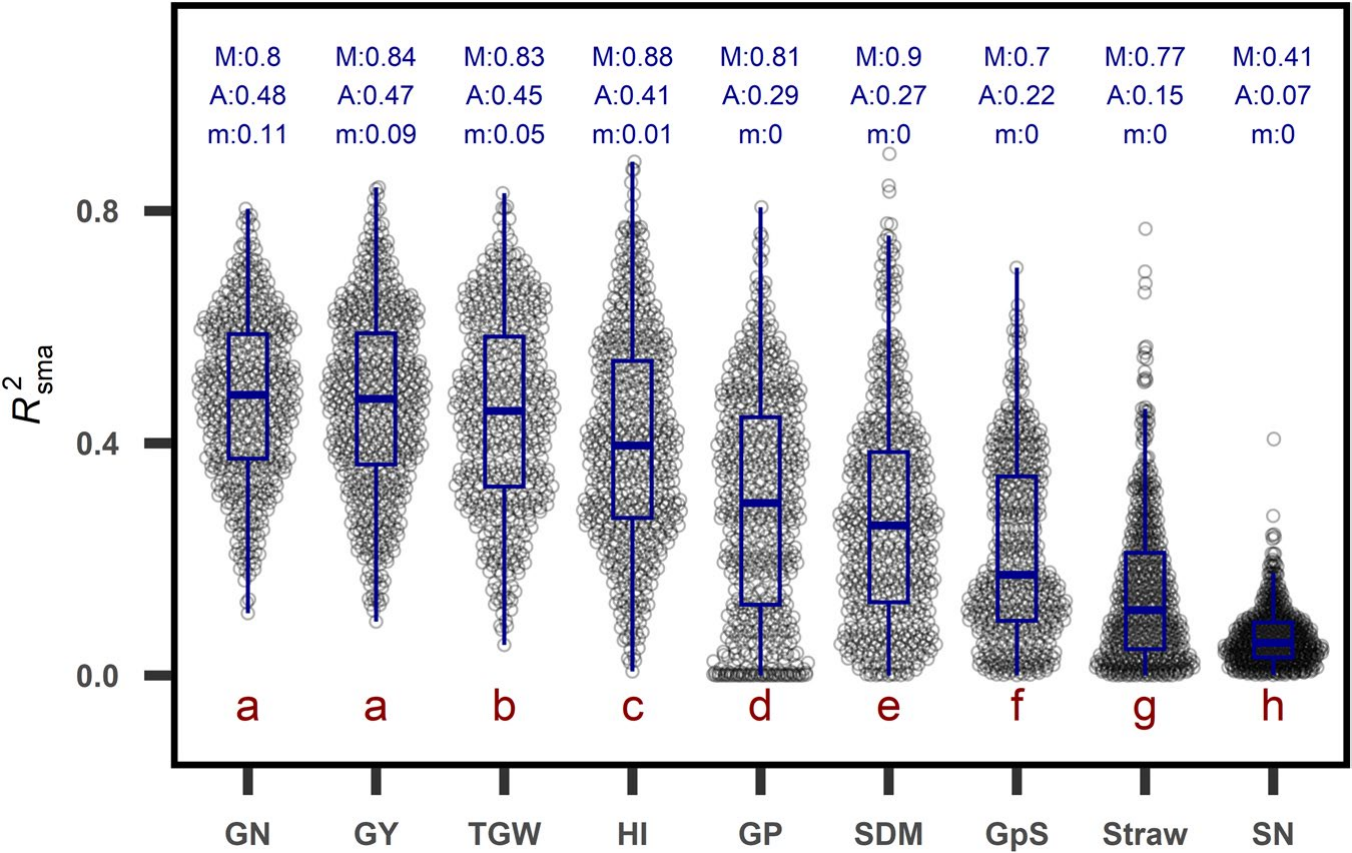
Trait consistency (R^2^_sma_) of nine agronomic traits across all combinations of growing conditions (Y/L/M). Each point represents the consistency of a trait between two Y/L/M. There are 9900 combinations in total, resulting from the permutation of two out of 45 Y/L/M. Blue letters above the boxplot denote three statistics of R^2^_sma_: M for maximum; A for average and m for minimum. Different dark red lowercase letters below denote statistical significance at level of alpha = 0.05 based on Fisher’s post hoc test following ANOVA. The abbreviation of nine traits are: grain yield (GY); grain number (GN); thousand grain weight (TGW); harvest index (HI); grain protein concentration (GP); above ground dry mass at maturity (SDM); grain per spike (GpS); straw dry mass at maturity (Straw) and spike number (SN).

R^2^ from standardized major axis regression (SMA; R^2^_sma_) was used to evaluate the consistency of traits between growing conditions (Y/L/M). R^2^_sma_ for grain yield range widely, spanning from 0.09 to 0.84, with an average of 0.47 (Fig. 4). In other words, on average, yield in one growing condition explained less than 50% of variation in yield in another growing conditions. Unexpectedly, although grain yield is the most complex trait, it showed the highest R^*2*^_sma_ together with grain number. Average R^*2*^_sma_ in TGW was at a similar level but significantly lower than that in grain yield. Above ground and straw dry mass at maturity, spike number and grain number per spike had low consistency (average R^*2*^_sma_ < 0.22), especially straw dry mass at maturity (R^*2*^_sma_ = 0.15) and spike number (R^*2*^_sma_ = 0.07), suggesting the strongest GxExM effects on tillering and canopy development.

**Fig. 5:**
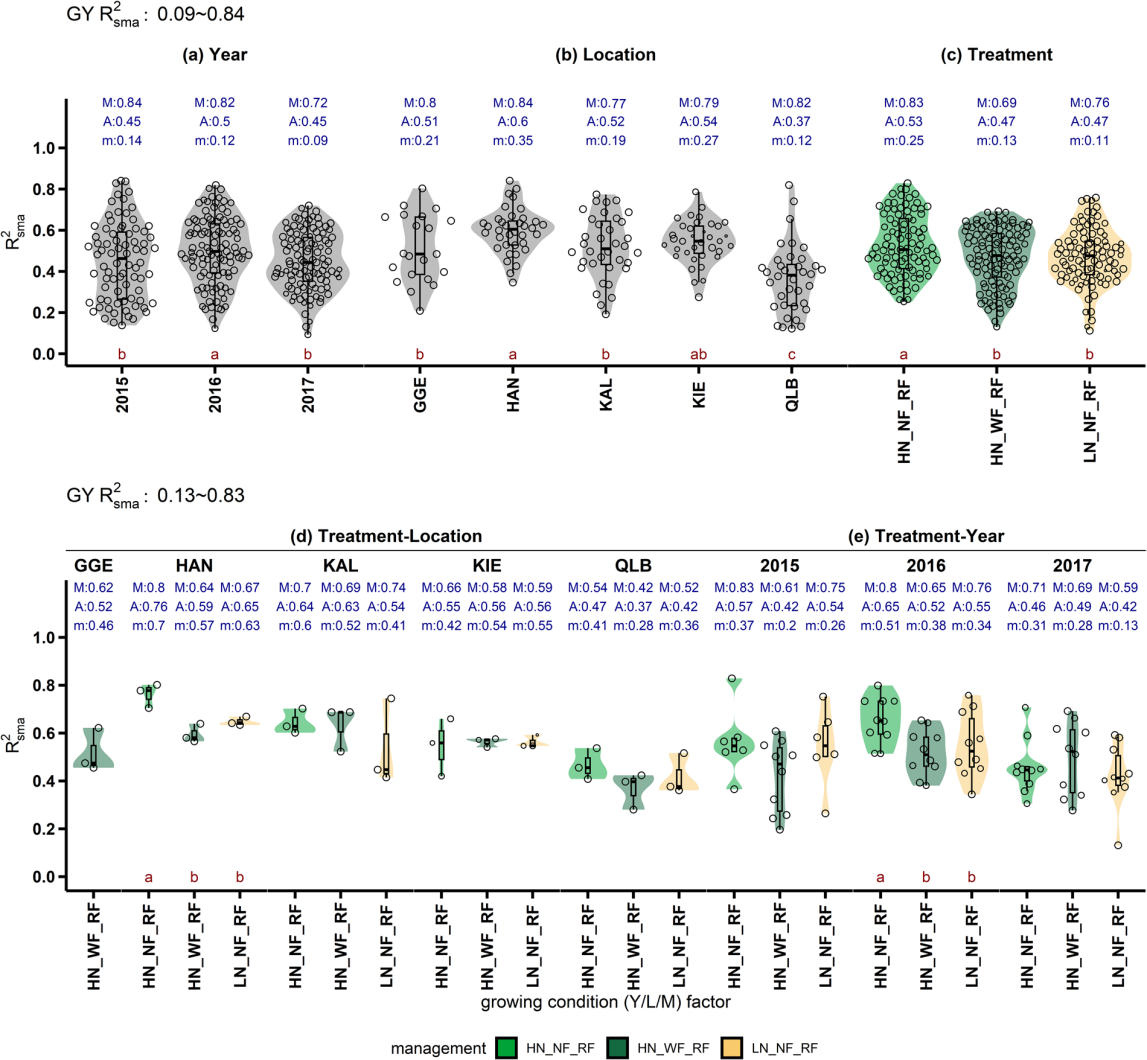
Consistency (R^2^_sma_) of grain yield (GY) with five groupings: (**a**) year, (**b**) location, (**c**) management, (**d**) management-location and (**e**) management-year. Each point represents a R^2^_sma_ of a trait between two Y/L/M. Different lowercase letters denote statistical significance at level of *p* = 0.05 based on Fisher’s post hoc test following analysis of variance. Abbreviation for locations: Gross Gerau (GGE), Hannover (HAN), Klein Altendorf (KAL), Kiel (KIE), Quedlinburg (QLB), and Rauischholzhausen (RHH). Un-balanced nine managements comprise of three treatments: nitrogen fertilizer, fungicide application and water availability. Nitrogen treatments has two levels: high (HN: 220 kg N ha^−1^) and low (LN: 110 kg N ha^−1^). Fungicide treatment contains two application levels: with (WF) or without (NF) fungicide application. Water availability treatment in this analysis has one levels: rain-fed (RF).

Furthermore, the consistency (R^*2*^_sma_) of grain yield differed between years, locations, and managements. Interestingly, R^*2*^_sma_ showed the largest variations between locations (Fig. 5). On average, R^*2*^_sma_ was the highest in Hannover (60%) and the lowest in Quedlinburg (37%). Notably, grain yield was more consistent under the management HN_NF_RF (high nitrogen without fungicide in rain-fed, average R^*2*^_sma_ = 0.53) than HN_WF_RF (high nitrogen and fungicide under rain-fed, average R^*2*^_sma_ = 0.47) and LN_NF_RF (low nitrogen without fungicide under rain-fed, average R^*2*^_sma_ = 0.47). This indicates that plant protection is a management that increases GxE, due to the fact that, in our panel, the accumulation of genes for diseases resistance is an important results of the breeding history^12^, and if the contribution of these genes on yield is replaced by the plant protection, the genotypic characteristic is not fully exploited, therefore less consistent results.

### Crop growth models overlook environmental interactions of dry mass allocation in field conditions

The second validation is the extent to which the results from crop model simulations represent “real world” data. To achieve this, the Pearson correlation coefficient (*r*) between two agronomic traits (referred to as trait-trait correlation) in the field was compared with the trait-trait correlation simulated by the well-calibrated crop simulation model APSIM-wheat (doi: 10.5281/zenodo.7569104)^21–23^. As examples, we selected two locations (Hannover and Kiel) from one management scenario (high nitrogen and with fungicide under rain-fed condition; HN_WF_RF), where the maximum number of directly comparable traits to APSIM-wheat can be found. Note that each location also has a different number of traits measured. The analysis of trait-trait correlations encompassed eleven traits (Table 2): grain number (GN), grain protein concentration (GP), grain yield (GY), harvest index (HI), thousand grain weight (TGW), radiation use efficiency (rue), leaf area index (LAI), light extinction coefficient (k), flowering time (FT), maturity time (MT), and straw dry mass at maturity (Straw). In APSIM-wheat, radiation use efficient (*rue*) and light extinction coefficient (*k*) are input parameters that can be varied between simulations and the rest of the traits are simulated outputs. For each available pair of traits, *r* was calculated for both the simulation and the MET dataset.

**Fig. 6:**
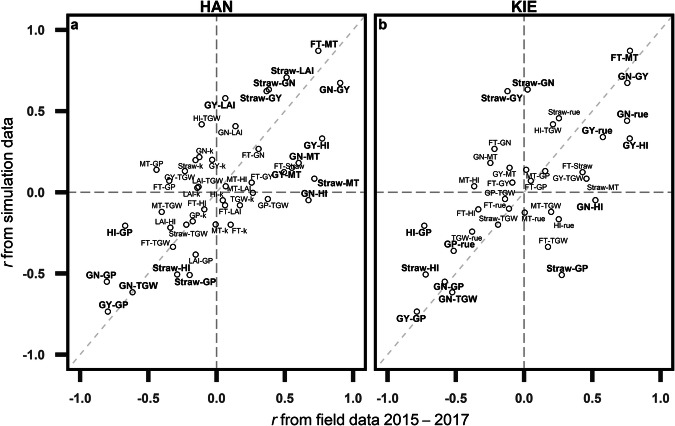
Comparison of trait-trait correlations between field experiments and crop model simulations. Field dataset from three consecutive years (2015–2017) under high nitrogen and fungicide application in rain-fed treatment (HN_WF_RF) from (**A**) Hannover and (**B**) Kiel was used. Simulation dataset comes from previous publications (doi: 10.5281/zenodo.7569104)^21,22^. Each point represents the Pearson correlation coefficient (*r*) between two traits observed in the field experiment (x-axis) and in the simulations of APSIM-wheat (y-axis). The diagonal dashed line represents a one-to-one line and the distance of a point to the one-to-one line represents the similarity of *r* between field and simulation. Abbreviation of ten traits are: flowering time (FT); harvest index (HI); light extinction coefficient (k); leaf area index (LAI); maturity time (MT); grain number (GN); grain protein concentration (GP); grain yield (GY); radiation use efficiency (rue); straw dry mass at maturity (Straw) and thousand grain weight (TGW). The trait-trait combinations were bolted if their distances to one-to-one line below 0.09 and both absolute value of x and y larger than 0.5.

To validate whether crop models considering GxExM interactions correctly represent field observations, we showed pairwise correlations among traits (trait-trait correlations) between simulations and detailed field observations at two locations (Hannover and Kiel). In general, three relationships aligned well between field observations and simulations (Fig. 6): (1) a positive correlation was observed between grain number and grain yield (both locations *r* > 0.75; simulations: *r* = 0.67); (2) a negative correlation was observed between grain yield and grain protein concentration (both locations *r* < −0.79; simulations: *r* = −0.74); (3) a negative correlation was observed between grain number and thousand grain weight (both locations *r* < −0.53; simulations: *r* = −0.61). Consistency in these well-known trait-trait correlations showcases the ability of the APSIM-wheat model to represent relationships between yield components. However, two trait-trait correlations from field observations are weak or missing in the simulation: (1) a positive correlation was observed between straw dry mass at maturity and maturity time (Hannover: *r* = 0.71; Kiel: *r* = 0.45; simulation: *r* = 0.08), indicating the missing link of phenology and the growth of straw (an indicator of canopy volume) in the APSIM-wheat model. Furthermore, (2) a positive correlation was found between grain number and harvest index (Hannover: *r* = 0.67; Kiel: *r* = 0.52; simulation: *r* = −0.04), indicating that the allocation of dry mass to straw and grains should be re-examined in the crop model.

Correlations related to straw dry mass showed contrasts between locations and were frequently inconsistent between field and simulation (Fig. 6). For instance, simulated results overestimated the positive correlation between grain yield and straw dry mass at maturity (Hannover: *r* = 0.37; Kiel: *r* = −0.12; simulation: *r* = 0.62). Additionally, simulated results overestimated the negative correlation between grain protein and straw dry mass at maturity (Hannover: *r* = −0.2; Kiel: *r* = 0.28; simulation: *r* = −0.5). Together, these results indicate a potential improvement of crop models by better considering the canopy development and dry mass allocation. Extensive MET trait datasets like the one described here are essential to achieve this.

### Breeding progress in straw dry mass at maturity, TGW and harvest index explain 48% of grain yield improvement

Since the data were collected to estimate breeding progress of different agronomic traits in winter wheat, the third validation showcased to which extent breeding progress (BP) in agronomic traits contributed to the BP in grain yield. BP was defined as the slope from simple linear regression between BLUEs of cultivars and their year of release. Analysis was conducted on a subset of 191 cultivars representing the breeding history of winter wheat in Germany between 1963 and 2013^10^. The agronomic traits (Table 2) include grain per spike (GpS), harvest index (HI), spike number (SN), thousand grain weight (TGW) and straw dry mass at maturity (Straw). BP analysis was conducted to all combinations (all) of Y/L/M or single grouping of Y/L/M by a multi-linear regression (3):

3 $${{\rm{BP}}}_{{\rm{GY}}}={{\rm{BP}}}_{{\rm{TGW}}}+{{\rm{BP}}}_{{\rm{HI}}}+{{\rm{BP}}}_{{\rm{straw}}}+{{\rm{BP}}}_{{\rm{GpS}}}+{{\rm{BP}}}_{{\rm{SN}}}+{\rm{\varepsilon }}$$

where all regressors of breeding progress are assumed to have fixed effect, and the error term is ε. With the regression model, we further quantify the relative importance of each regressor from multi-linear regression using R package *relaimpo*^24^.

**Fig. 7:**
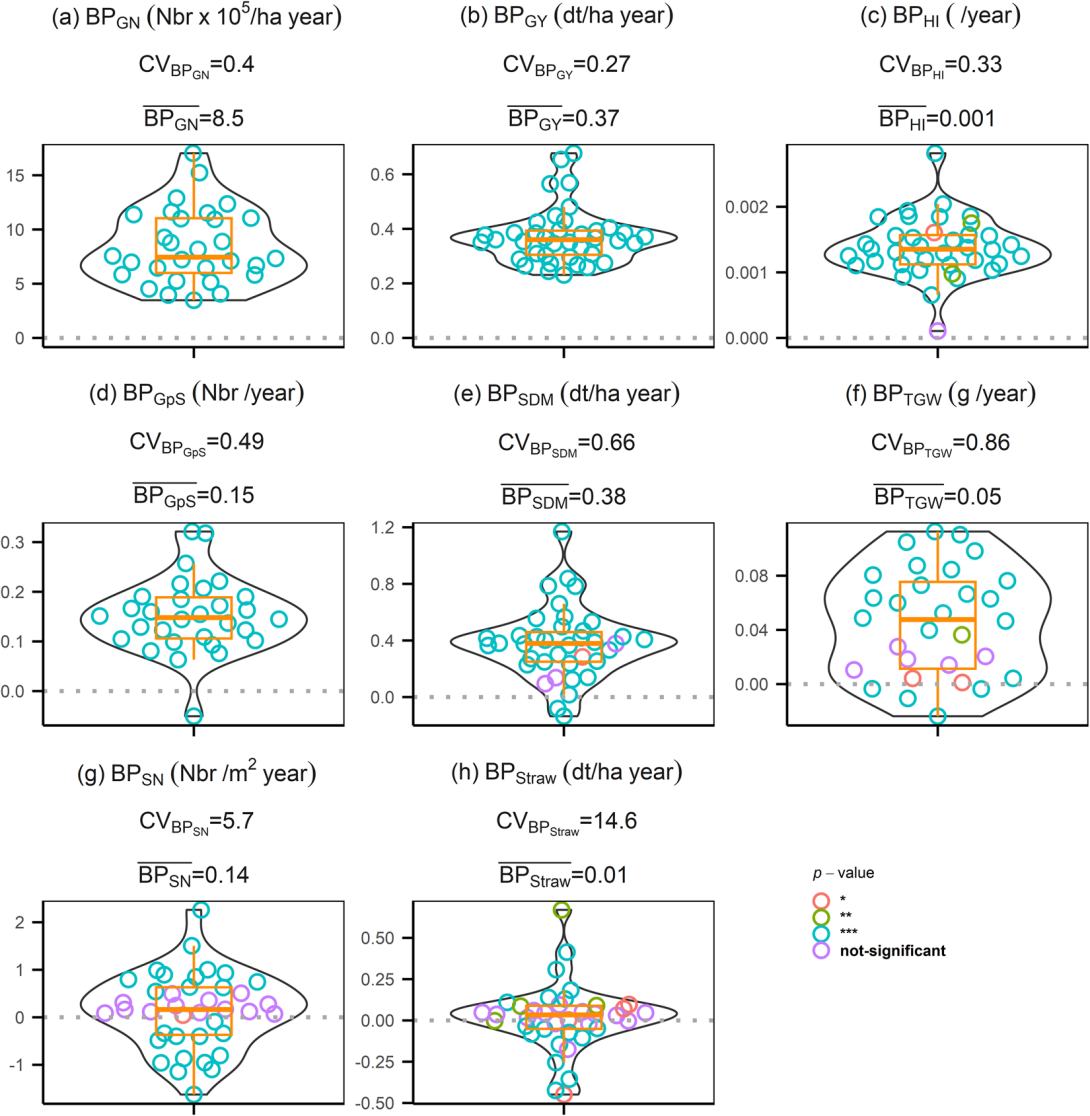
Distribution of breeding progress (BP) of eight agronomic traits from all combinations of growing conditions (Y/L/M). (a-h) Abbreviation of eight traits: straw dry mass at maturity (Straw), spike number (SN), thousand grain weight (TGW), shoot dry mass at maturity (SDM), grain per spike (GpS), grain number (GN), harvest index (HI), grain yield (GY). Unit abbreviations: Nbr stands for number; year stands for difference in year of release between genotypes. Colours and stars symbols refers to significance level of p-value of each term in (5): * and red refers to significance at 5% level; ** and green refers to significance at 1% level; *** and blue refers to significance at 0.1% level; purple refers to not significant with p-value larger than 5% level. CV_BPtrait_ stands for coefficient of variation; BP_trait_ over bar stands for mean BP_trait_.

Breeding progress (BP) of grain yield and six other agronomic traits (Table 2) varied largely between years, locations and managements and showed contrasting distributions across 40 growing condition (Y/L/M) (Fig. 7). Grain number, grain yield and harvest index have all BP values above zero, while the most inconsistent traits, namely spike number and straw dry mass at maturity (Fig. 4), have negative BP values in 13 (33%) and 17 (42%) of the 40 growing conditions, respectively. Breeding progress for grain yield (BP_GY_) showed three-fold differences between growing conditions, ranging from 0.23 to 0.68 (dt/ha year) with average BP_GY_ = 0.37 (dt/ha year). In general, BP values close to 0 are more likely to show a higher p-value of regression.

**Fig. 8:**
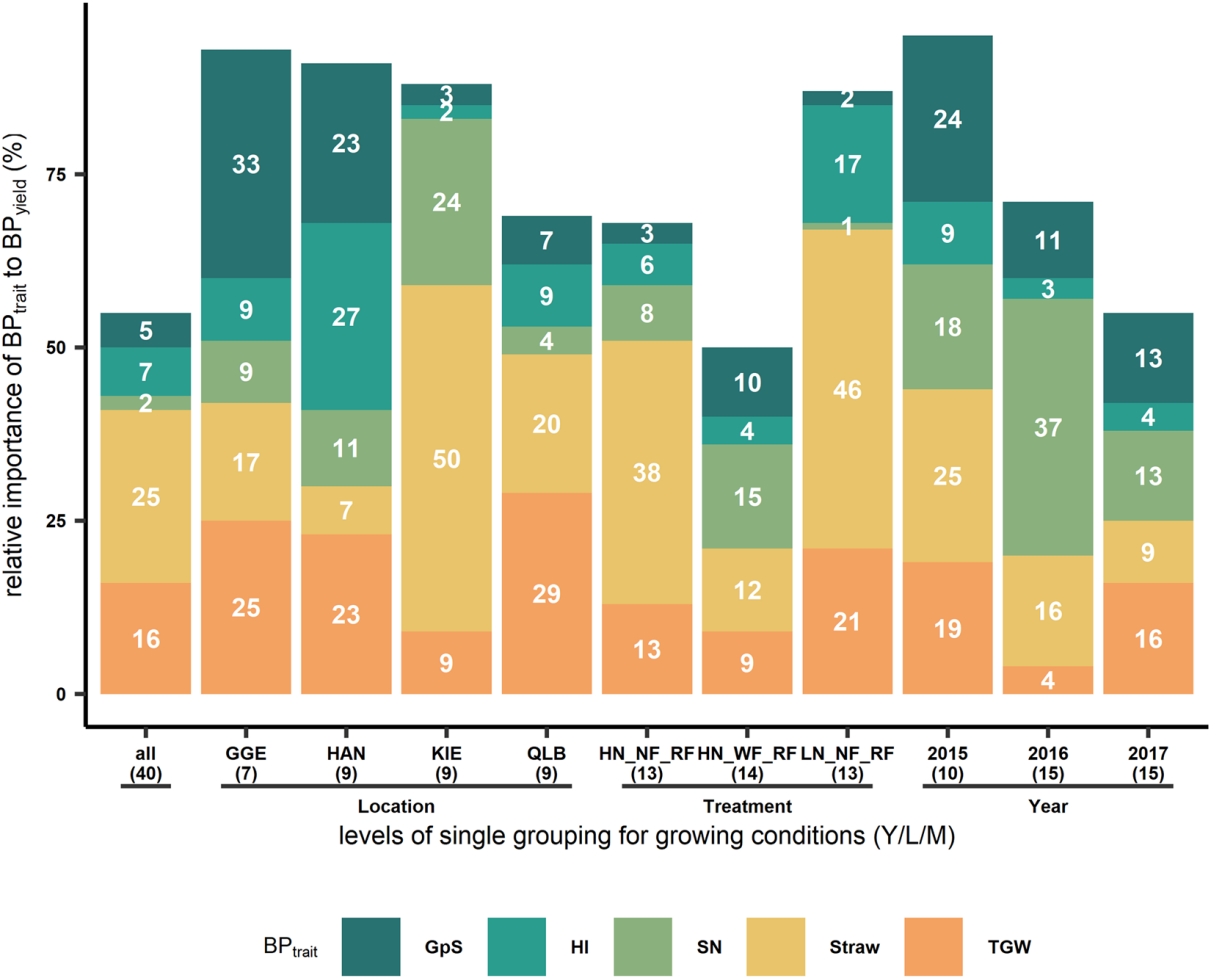
Multi-linear regression analysis breeding progress (BP) in five traits to the BP of grain yield from no groupings (all) or single grouping of growing condition (Y/L/M). Stacked bar plot represent the relative importance of each trait in (4). Number in bracket refers to the number of observation for each level. Abbreviations of five traits are: grain per spike (GpS); harvest index (HI); spike number (SN); straw dry mass at maturity (Straw); total fungal infection area (TFI); and thousand grain weight (TGW). X-axis: no grouping (all: all growing conditions) and levels from single grouping of growing conditions.

The contribution of BP for five agronomic traits (Table 2) to BP for grain yield can be validated by multi-linear regression (3) with or without grouping of growing conditions. In most cases the regression coefficient (*β*) was not significant (*p*-value > 0.05) and showed no pattern across the grouping (Table 3). In cases without grouping (all), BP_Straw_, BP_TGW_ and BP_HI_ were significant regressors, which collectively explained 48% of the R^2^ in BP_GY_ (Fig. 8). Note that the result from relative importance should be considered together with the *β*. Positive *β* of these three traits suggested that growing conditions stimulating stronger straw growth, higher grain per spike and heavier grain of the modern cultivars led to higher BP_GY_. Non-significance in *β* could be related to the low number of observations (number in brackets in

**Table 3:** Coefficient of regressors in multi-linear regression (3) for breeding progress of grain yield with or without grouping (all) of growing conditions.

Trait	HN_NF_RF	HN_WF_RF	LN_NF_RF	GGE	HAN	KIE	QLB	2015	2016	2017	all
BP _(Intercept)_	0.25	0.14	−0.1	0.2	−0.21	0.25	0.19	0.42	0.19 ^*^	0.28 ^**^	0.12 ^*^
BP_GpS_	0.07	0.58	0.09	−1.4	2.8	−0.73	0.15	1.7	0.66 ^*^	0.62	0.31
BP_HI_	49.6	70.5	270.3 ^**^	181.5	82.3	134.9	102.2	−142	−30.5	−83.5	101.1 ^*^
BP_SN_	−0.03	−0.02	0.005	−0.03	0.02	−0.04	−0.02	−0.06	0.13 ^*^	0.02	−0.003
BP_Straw_	0.59 ^*^	0.27	0.72 ^**^	0.66	−0.48	1.1	0.21	0.66 ^*^	0.1	0.04	0.36 ^***^
BP_TGW_	0.72	0.94	0.4	1.2	1.6	0.05	0.92	−0.35	1	1.8	0.94 ^*^

Stars symbol refers to significance level of *p*-value of each term in (5): * significant at 5% level; ** significant at 1% level; *** significant at 0.1% level.

Abbreviation for locations: Gross Gerau (GGE), Hannover (HAN), Klein Altendorf (KAL), Kiel (KIE), Quedlinburg (QLB), and Rauischholzhausen (RHH). Un-balanced nine managements comprise of three treatments: nitrogen fertilizer, fungicide application and water availability. Nitrogen treatments has two levels: high (HN: 220 kg N ha^−1^) and low (LN: 110 kg N ha^−1^). Fungicide treatment contains two application levels: with (WF) or without (NF) fungicide application. Water availability treatment in this analysis has one levels: rain-fed (RF).

Figure 8). which could reduce the degree of freedom or the co-linearity among regressors.

### TFI, stability in HI, and GpS contributes to 77% of the yield stability

The last validation of the dataset involved nine stability indices (SI) and showed the contribution of stability in five agronomic traits plus one pathogen trait to the stability of grain yield (GY). The five agronomic traits (Table 2) were grain protein (GP), grain per spike (GpS), harvest index (HI), spike number (SN), thousand grain weight (TGW), straw dry mass at maturity (Straw). The single pathogen trait considered in this case was the total fungal infection area (TFI). Nine SI including both static and dynamic concepts of stability were chosen: coefficient of determination (*r*^2^_i_), coefficient of regression (*b*_i_), deviation mean squares (*s*^2^_di_), ecovalence (*W*_i_), environmental variance (*S*^2^_xi_), genotypic stability (*D*^2^_i_), genotypic superiority measure (*P*_i_), stability variance (*σ*^2^_i_), variance of rank (*S*_i4_). Each SI was calculated for each genotypes of a trait. For each SI, a multi-linear regression was implemented (4):

4 $${{\rm{SI}}}_{{\rm{GY}}}={{\rm{SI}}}_{{\rm{TGW}}}+{{\rm{SI}}}_{{\rm{HI}}}+{{\rm{SI}}}_{{\rm{straw}}}+{{\rm{SI}}}_{{\rm{GpS}}}+{{\rm{SI}}}_{{\rm{SN}}}+{\rm{TFI}}+{\rm{\varepsilon }}$$

where all regressors of stability indices are assumed to have fixed effect, and the error term is ε. Similar to technical validation III, the relative importance of each regressor from multi-linear regression was quantified using R package *relaimpo*^24^ and stability index was calculated using R package *toolStability*^25^.

To validate the contribution of stability in seven agronomic traits to the yield stability, stability indices (SI) were calculated and multi-linear regression analyses (4) were conducted. As shown in in Table 4, the regression coefficient (*β*) showed significant and positive contribution of SI_TGW_ and SI_HI_ to SI_GY_. Among nine SI, genotypic superiority index (*P*_*i*_) for yield was best explained by the *P*_*i*_ of seven traits and showed significance in *β* for every trait considered.

**Table 4:** Coefficient of seven regressors from multi-linear regression (4) for nine stability of grain yield of 220 genotypes.

Trait	b_i_	D_i_^2^	P_i_	r_i_^2^	S_xi_^2^	S_di_^2^	S_i_4	σ_i_^2^	W_i_
SI_(Intercept)_	0.06	−3.5	−24.7 ^***^	−0.2 ^**^	0.29	−0.59	−15.7 ^*^	−0.87	−5
SI_GP_	0.27 ^***^	0.77	−1.3 ^**^	0.16 ^*^	1.8 ^***^	0.31	−0.02	0.46	0.4
SI_GpS_	0.16 ^***^	0.41 ^***^	0.64 ^***^	0.01	0.41 ^***^	0.22 ^**^	0.18 ^**^	0.19 ^*^	0.22 ^*^
SI_HI_	0.48 ^***^	94.5 ^***^	109.3 ^***^	0.39 ^***^	79.2 ^***^	82.6 ^***^	0.46 ^***^	74.7 ^***^	83.3 ^***^
SI_SN_	0.02	0.002	0.04 ^***^	0.08 ^***^	0.004	0.008	0.12	0.01	0.009
SI_Straw_	−0.2 ^***^	−0.05	0.19 ^***^	0.004	−0.08 ^**^	−0.009	0.18 ^*^	0.008	0.005
SI_TGW_	−5e-04	0.4 ^**^	0.28 ^***^	−6e-04	0.05 ^*^	0.05 ^*^	0.11	0.05 ^*^	0.27
TFI	0.22 ^***^	0.96 ^***^	0.84 ^***^	0.58 ^***^	0.67 ^***^	1.2 ^***^	0.29 ^***^	1.3 ^***^	1.5 ^***^

Stars symbol refers to significance level of *p*-value of each term in (5): * significant at 5% level; ** significant at 1% level; *** significant at 0.1% level.

Nine stability indices (SI): coefficient of determination (*r*^2^_i_), coefficient of regression (*b*_i_), deviation mean squares (*S* ^2^_di_), ecovalence (*W*_i_), environmental variance (*S*^2^_xi_), genotypic stability (*D*^2^_i_), genotypic superiority measure (*P*_i_), stability variance (*σ*^2^_i_), variance of rank (*S*_i4_). Abbreviations of seven traits: grain protein concentration (GP); grain per spike (GpS); harvest index (HI); spike number (SN); straw dry mass at maturity (Straw); total fungal infection area (TFI); and thousand grain weight (TGW).

**Fig. 9:**
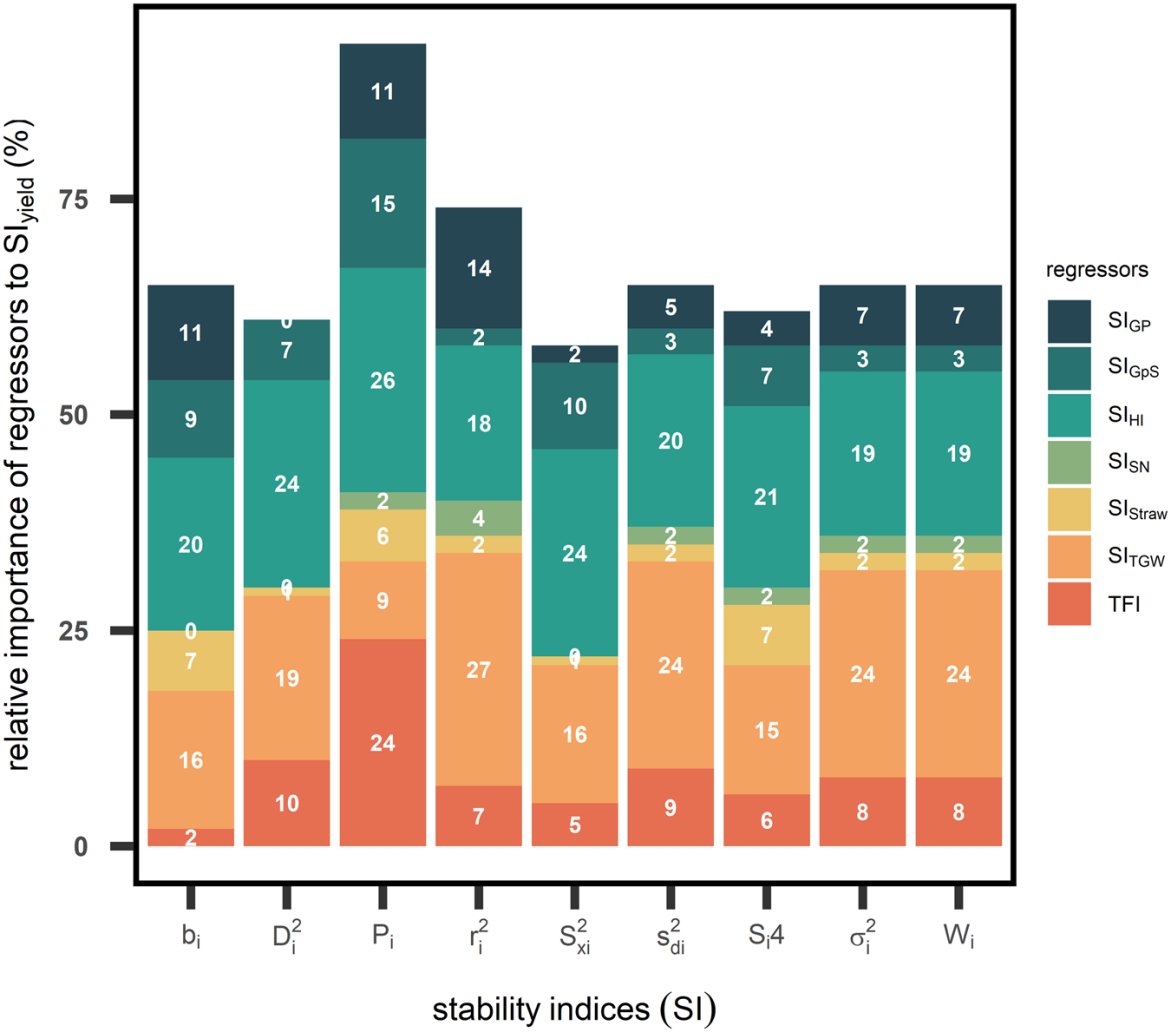
Multi-linear regression analysis of stability in six traits (SI_trait_) to stability of grain yield using nine stability indices (SI). Stacked bar plot represents relative importance of each trait in (5). Abbreviations of seven traits: grain protein concentration (GP); grain per spike (GpS); harvest index (HI); spike number (SN); straw dry mass at maturity (Straw); total fungal infection area (TFI); and thousand grain weight (TGW). Nine SI: coefficient of determination (*r*^2^_i_), coefficient of regression (*b*_i_), deviation mean squares (*S* ^2^_di_), ecovalence (*W*_i_), environmental variance (*S*^2^_xi_), genotypic stability (*D*^2^_i_), genotypic superiority measure (*P*_i_), stability variance (*σ*^2^_i_), variance of rank (*S*_i4_).

The contribution of regressors to R^2^ varied between SI (Fig. 9), ranging between 61% (variance of rank; *S*_i4_) to 94% (*P*_i_). SI_TGW_ and SI_HI_ were of most important traits and contributed collectively at least 36% to R^2^ across SI. In the case of *P*_i_, 77% of R^2^ of SI_GY_ could be explained by the three main contributors: total fungi infection area (TFI), SI_HI_ and SI_Gps_. Interestingly, the stability of the least consistent traits - spike number and straw (Fig. 4) - explained together less than 6% of SI_GY_ (Fig. 9). Furthermore, we showed that the relative importance of the stability of a trait depended on SI. For instance, TFI explain from 1.5% (coefficient of regression; *b*_i_) to 26% (*P*_*i*_) of R^2^ in SI in grain yield.

## Supplementary information

supplementary table

## Data Availability

The data were processed in R (version 4.3.2). The code to reproduce the results in this publication is publicly available at https://github.com/tillrose/BRIWECS_Data_Publication (pre-processing and visualization) and https://github.com/Illustratien/Scientific_Data_Analyis (technical validation I–IV). Both codes are subject to the MIT license (https://opensource.org/license/mit).
